# 

**Published:** 1884-06

**Authors:** 


					﻿JTJJSTE 1884..
OLD SERIES
Vol. XXV
1855 O'
NEW SERIES
Vol. I.—No. 4./
MM®
lEDIGAL^RGffi
Journal

?>by'
W.F.WESTMOREtTAND,
H.V.M.MILLER.M.O.LL.D.J^
JAMES A.GRAY, M.D."
Published By james p.harrison&cz
HP	TflTLANTA ,GA.
SUBSCRIPTION: 2.50 A YEAR.
				

